# Autophagy and autophagy signaling in Epilepsy: possible role of autophagy activator

**DOI:** 10.1186/s10020-023-00742-2

**Published:** 2023-10-25

**Authors:** Naif H. Ali, Hayder M. Al-kuraishy, Ali I. Al-Gareeb, Saud A. Alnaaim, Athanasios Alexiou, Marios Papadakis, Hebatallah M. Saad, Gaber El-Saber Batiha

**Affiliations:** 1 Department of Internal Medicine, Medical College, Najran university, Najran, Saudi Arabia; 2 Department of Clinical Pharmacology and Medicine, College of Medicine, ALmustansiriyia University, P.O. Box 14132, Baghdad, Iraq; 3 Clinical Neurosciences Department, College of Medicine, King Faisal University, Hofuf, Saudi Arabia; 4 Department of Science and Engineering, Novel Global Community Educational Foundation, Hebersham, NSW 2770 Australia; 5 AFNP Med, Wien, 1030 Austria; 6 Department of Surgery II, University Hospital Witten-Herdecke, University of Witten-Herdecke, Heusnerstrasse 40, 42283 Wuppertal, Germany; 7 Department of Pathology, Faculty of Veterinary Medicine, Matrouh University, Matrouh, Matrouh, 51744 Egypt; 8 Department of Pharmacology and Therapeutics, Faculty of Veterinary Medicine, Damanhour University, Damanhour, AlBeheira, 22511 Egypt

**Keywords:** Neurodegeneration, Epilepsy, Seizure, Autophagy, Autophagy inducers

## Abstract

Autophagy is an explicit cellular process to deliver dissimilar cytoplasmic misfolded proteins, lipids and damaged organelles to the lysosomes for degradation and elimination. The mechanistic target of rapamycin (mTOR) is the main negative regulator of autophagy. The mTOR pathway is involved in regulating neurogenesis, synaptic plasticity, neuronal development and excitability. Exaggerated mTOR activity is associated with the development of temporal lobe epilepsy, genetic and acquired epilepsy, and experimental epilepsy. In particular, mTOR complex 1 (mTORC1) is mainly involved in epileptogenesis. The investigation of autophagy’s involvement in epilepsy has recently been conducted, focusing on the critical role of rapamycin, an autophagy inducer, in reducing the severity of induced seizures in animal model studies. The induction of autophagy could be an innovative therapeutic strategy in managing epilepsy. Despite the protective role of autophagy against epileptogenesis and epilepsy, its role in status epilepticus (SE) is perplexing and might be beneficial or detrimental. Therefore, the present review aims to revise the possible role of autophagy in epilepsy.

## Introduction

Epilepsy is defined as a condition characterized by recurrent two or more epileptic seizures, unprovoked by any immediate identified cause (Thijs et al. 2019) that is an abnormal hyper-synchronous neuronal discharge from a defined brain region (Thijs et al. 2019). One seizure attack can occur in any subject that is not considered epilepsy, but investigations are reasonable to outline the original cause of the seizure (Fisher et al. 2022). Nevertheless, a history of two or more seizures is defined as epilepsy (Thijs et al. 2019). Furthermore, status epilepticus (SE) is defined as a seizure that persists for a sufficient length of time or is repeated frequently enough that recovery between attacks does not occur (Xu et al. 2004). Other findings showed that SE is a seizure that persists for 20 to 30 min, an estimate of the duration necessary to cause injury to central nervous system (CNS) neurons (Burman et al. 2022). The overall mortality rate among adults with SE is approximately 20%, and patients who have a first episode of SE are at substantial risk for future episodes and the development of chronic epilepsy (Xu et al. 2004; Burman et al. 2022). Epilepsy significantly burdens the quality of life of affected individuals and their families. Epilepsy is a chronic disease experienced by millions and a cause of substantial morbidity and mortality. Epilepsy affects about 1% of the general population internationally till 2020 (Miller et al. 2020). It has been reported that 80% of epileptic cases universally are in developing countries, and it is further common in the elderly (Kissani et al. 2020). In addition, 5–10% of old people have a seizure at the age of 80, increasing the chance of a second seizure by 40–50% (Cretin 2021).

Since the introduction of bromide as an anti-seizure drug in 1857, there has been an impressive expansion of clinically effective therapies in decreasing the frequency and severity of seizures in people with epilepsy. This class of symptomatic treatments is widely referred to as antiepileptic drugs (AEDs) (Orrego-GonzÁlez et al. 2020; Nasif et al. 2021; Komagamine et al. 2021). AEDs including phenobarbital and phenytoin were introduced to manage epilepsy in 1912 and 1938 respectively (Paprocka et al. 2019).

**Fig. 1:**
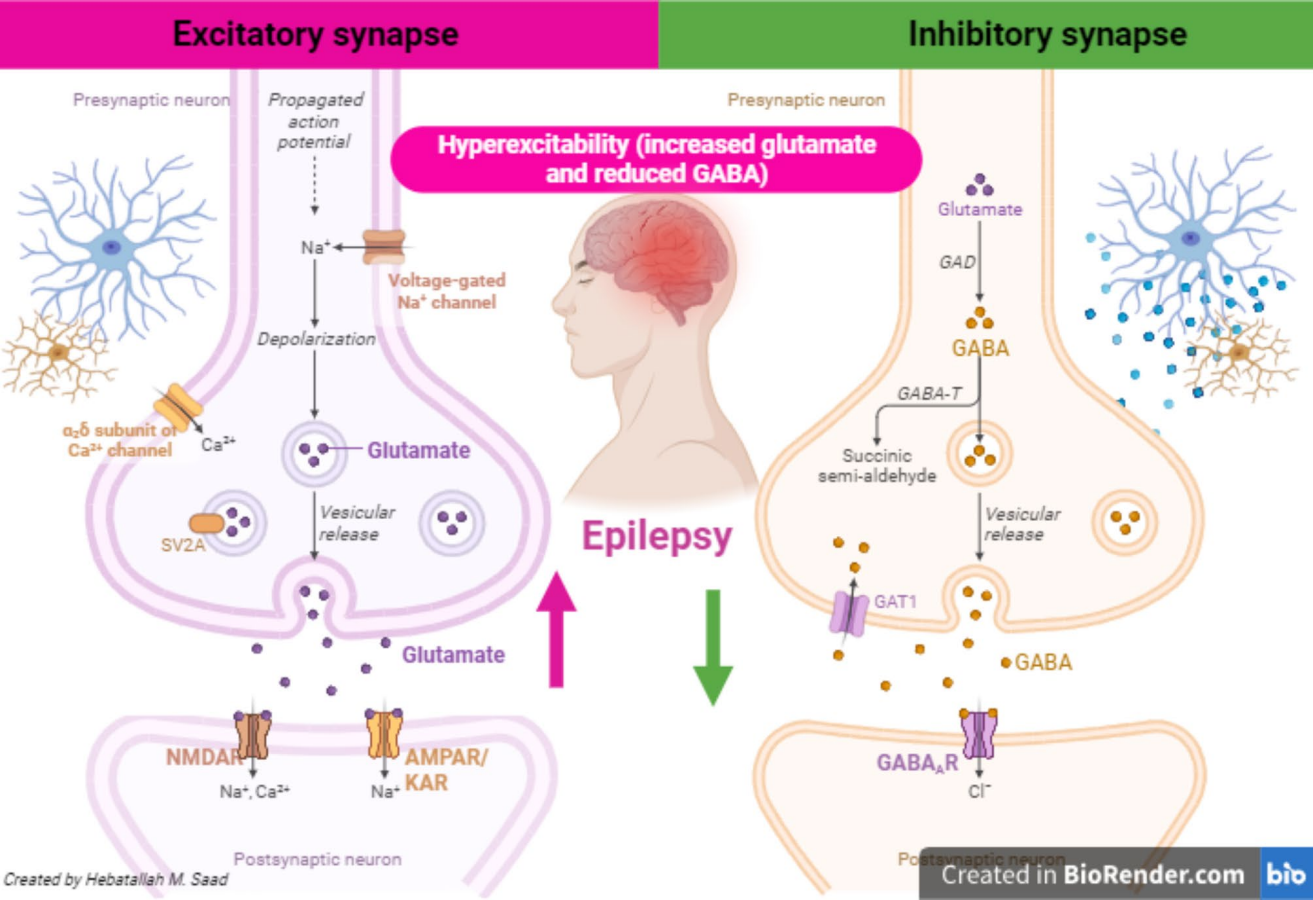
Pathophysiology of epilepsy. Decreasing inhibitory gamma-aminobutyric acid (GABA) and increasing excitatory glutamate persuade the progress and progression of epileptogenesis

The central mechanism of epileptic seizure is the epileptogenesis process which occurs due to an imbalance between inhibitory and excitatory neurotransmitters (Li et al. 2020a, b). Epileptogenesis is defined as the process of developing epilepsy a disorder characterized by recurrent seizures following an initial insult. Seizure incidence during the human lifespan is at its highest in infancy and childhood. As well, epileptogenesis is a chronic process that genetic or acquired factors can trigger, and that can continue long after epilepsy diagnosis (Li et al. 2020a, b). Decreasing inhibitory gamma-aminobutyric acid (GABA) and increasing excitatory glutamate favor the progression of epileptogenesis (Li et al. 2020a, b). The intention for such inequity is prominently unknown, though mutation of voltage-gated Na^2+^, Ca^2+^ and K^2+^ channels aggravates neuronal hyper-excitability and decreases seizure threshold (Pan et al. 2018). The mutation of the Na^2+^ channel gene SCN8A is accompanied by the development of epileptogenesis (Zaman et al. 2019). A comprehensive single-center dataset for *SCN8A* epilepsy that includes clinical, genetic, electrophysiologic, and pharmacologic data confirm a spectrum of severity and a variety of biophysical defects of Nav1.6 variants consistent with gain of channel function. Na + channel blockers in the treatment of *SCN8A* epilepsy may correlate with the effect of such agents on pathological Na + current observed in heterologous systems (Zaman et al. 2019). Therefore, mutation of voltage-gated Na^+^, associated neuronal hyper-excitability, and imbalance of excitatory/inhibitory circuit can reduce seizure threshold leading to the development of epilepsy (Fig. 1).

The causes of primary epilepsy are idiopathic (O’Neill et al. 2020). Nevertheless, secondary epilepsy is caused by diverse causes, including head trauma, brain infection, tumors, and neurodegenerative disorders (Steriade et al. 2020). Idiopathic generalized epilepsies (IGEs) affect about 1% of the population worldwide and are among the most common neurological disorders (Gesche and Beier 2022). IGEs are a group of presumably genetic epilepsies encompassing several clinical phenotypes such as childhood absence epilepsy and generalized epilepsy. In neuronal networks, either a gain of function of receptor channels mediating excitatory neurotransmission or a loss of function of receptor channels mediating inhibitory neurotransmission could impair the subtle balance of excitation and inhibition in the brain, thus, leading to disinhibition and seizures (Lehner et al. 2022). Genetic advances and functional characterizations of the pathophysiological alterations in receptor channel trafficking and function are changing our understanding of IGEs showed clear that genetic mutation of transmembrane ion channels is the underlying cause of many forms of human IGEs (O’Neill et al. 2020; Gesche and Beier 2022; Lehner et al. 2022). In IGEs, patients are asymptomatic between seizures. Radiological investigations are negative. Frequently, there is an overlap of IGEs, especially of those manifesting in later childhood and adolescence. Response to AEDs treatment and psychosocial prognosis are good. However, symptomatic (secondary) epilepsy and syndromes usually start in infancy or early childhood. In most children, several seizure types occur (Steriade et al. 2020). Electroencephalogram (EEG) discharges are less rhythmical and less synchronous than in idiopathic generalized epilepsies. There are neurological, neuropsychological, and radiological signs of diffuse cerebral disease. The only difference between cryptogenic and symptomatic syndromes is that in cryptogenic syndromes the presumed cause cannot be identified (Shellhaas et al. 2021).

It has been shown that dysregulation of autophagy is involved in the pathogenesis of epilepsy (Zheng et al. 2018; Ali et al. 2019). It has been suggested that autophagy alterations are present in epilepsy. In addition, rapamycin, a powerful autophagy inducer, strongly modulates a variety of seizure models and epilepsies. These findings were originally interpreted as the results of the inhibition exerted by rapamycin on the molecular complex named mammalian target of rapamycin (mTOR) (Ali et al. 2019; Giorgi et al. 2015). The present review features a brief introductory statement about the autophagy machinery and discusses the involvement of autophagy in seizures and epilepsies. An emphasis is posed on evidence addressing both pros and cons making it sometimes puzzling and sometimes evident, the role of autophagy in the epileptic brain. Therefore, the present review aimed to revise the potential role of autophagy in epilepsy, and how modulators of autophagy affect epileptogenesis.

## Autophagy overview

**Fig. 2:**
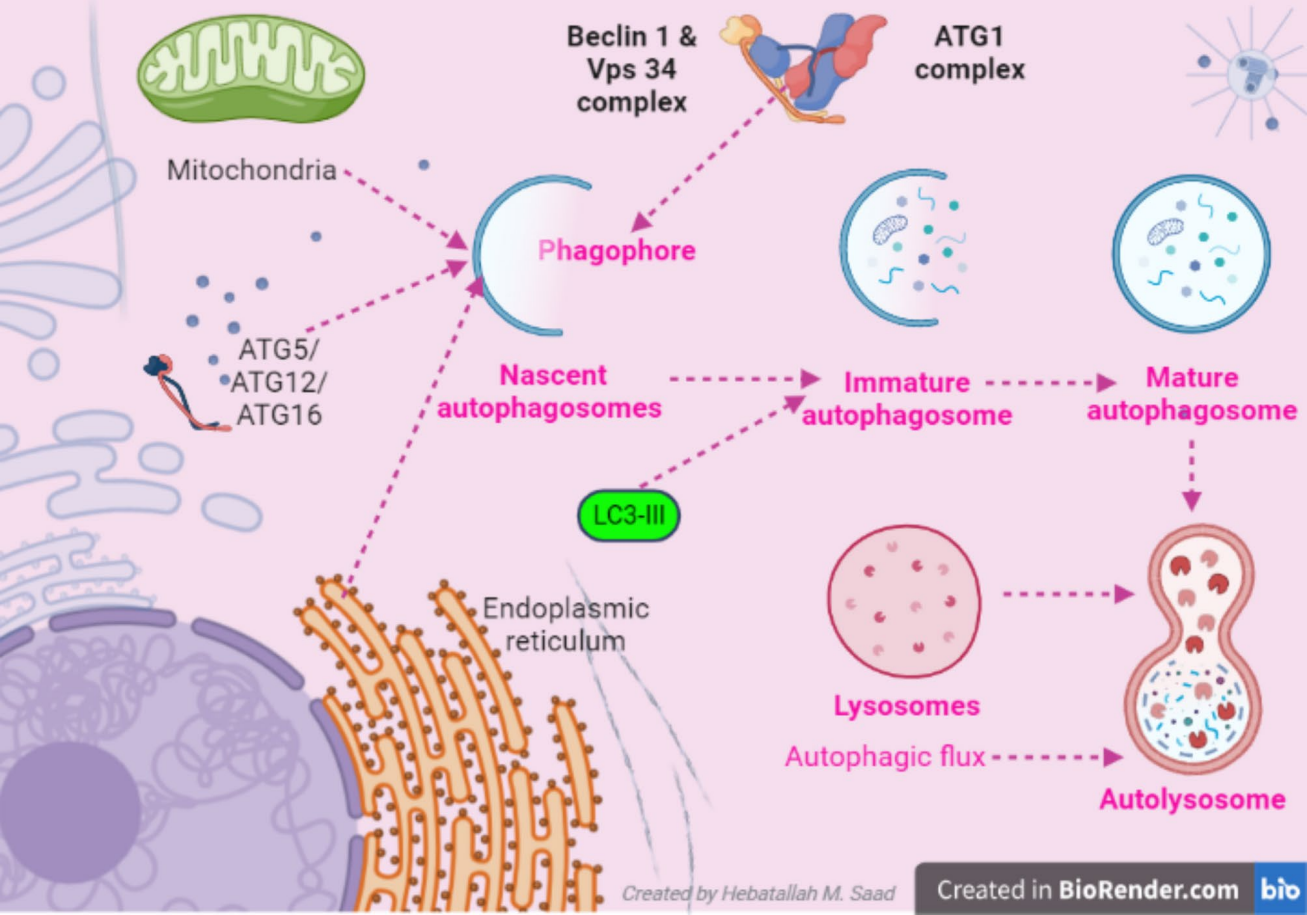
**Pathway of autophagy.** ATG1 complex, Beclin 1 and vacuolar sorting protein 34 (Vps34) triggered the formation of nascent autophagosomes from phagophores then with the assistance of LC3 form immature autophagosomes which are converted to mature autophagosomes. With the assistance of autophagic flux, mature autophagosomes together with lysosomes form autolysosomes

Autophagy is a major intracellular pathway for the degradation and recycling of long-lived proteins and cytoplasmic organelles. Li et al. ([Bibr CR12][Bibr CR23], 2023). Autophagy includes micro-autophagy, macro-autophagy and chaperone-mediated autophagy (CMA) (Udoh et al. 2022). Commonly, macro-autophagy is called autophagy, in which cytoplasmic debris is engulfed within multi-membrane vesicles called autophagosomes that delivered their contents to lysosomes for degradation (Udoh et al. 2022; Broggi et al. 2020). Therefore, autophagy contributes to renewing cell constituents by using cytoplasmic macro-molecules to form energy-rich compounds according to the bio-energetic demands (Ren et al. 2022). Different autophagy-related proteins ATG7, ATG12, ATG16, and LC3 are involved in forming autophagosomes from phagophores which are triggered by ATG1 complex, Beclin 1 and vacuolar sorting protein 34 (Vps34) complex (Sascha and Dorotea 2020). Nascent autophagosome with the assistance of LC3 forms immature autophagosomes which are converted to mature autophagosomes (Puri and Rubinsztein 2020). With autophagic flux’s assistance, mature autophagosomes and lysosomes form autolysosomes (Wang et al. 2023). Autophagic vacuole formation is also activated as an adaptive response to a variety of extracellular and intracellular stimuli, including nutrient deprivation, hormonal or therapeutic treatment, bacterial infection, aggregated and misfolded proteins and damaged organelles (Sascha and Dorotea 2020). Mediators of class I and class III PI3 kinase signaling pathways and trimeric G proteins play major roles in regulating autophagosome formation during the stress response (Puri and Rubinsztein 2020) (Fig. 2).

### Autophagy signaling

#### mTOR signaling pathway

Different autophagy signals are involved in the formation of autophagosomes. The mTOR is the main negative regulator of autophagy (Caza et al. 2022). It has been observed that both starvation and mTOR inhibitor rapamycin inhibit mTOR with subsequent activation of autophagy via activation of Atg13, ULK1, and ULK2 (Nowosad et al. 2020). Moreover, starvation-induced autophagy is mediated by attenuating the interaction between apoptosis-related protein B cell lymphoma 2 (Bcl-2), basal cell lymphoma extra-large (Bcl-X_L_) and Beclin-1 (Atg6) (Kadry et al. 2022). Also, starvation via activation of c-Jun N-terminal kinase-1 inhibits the interaction between Bcl-2 and Beclin-1 leading to the induction of autophagy (Kadry et al. 2022).

Growth factors like insulin-like growth factor (IGF) through activation of IGF receptors (IGFRs) promote the activity of tyrosine kinase and Aκt leading to activation of the mTOR pathway and inhibition of autophagy (Sepúlveda et al. 2022). Mild starvation also inhibits the mTOR pathway, but the kinase activity remains unaffected leading to acceleration of fusion between the autophagosome and lysosomes (Golpour et al. 2022). However, when the nutrients are replenished the mTOR pathway is inhibited with a significant reduction of autophagosomes (Golpour et al. 2022). Moreover, the p53 gene negatively and positively regulates the mTOR pathway and autophagy (Tong et al. 2020). Genotoxic and oncogenic stress activate p53 which induces activation of adenosine monophosphate protein kinase (AMPK) and phosphate and tensin homology (PTEN) which inhibits Aκt signaling (Wang et al. 2021). Inhibition of p53 also activates autophagy (Wang et al. 2019a, b, c). AMPK induces autophagy by inhibition of the mTOR pathway, or through activation of U1k1 (Saikia and Joseph 2021). Furthermore, inhibition of inositol monophosphatase (IMPase) independent of the mTOR pathway induces autophagy (Yang et al. 2021). Supporting this notion, the inhibition of IMPase by carbamazepine, lithium, and valproate improves autophagy in various neuropsychiatric disorders (Schmukler and Pinkas-Kramarski 2020).

#### IP3 signaling pathway

Inositol 1,4,5-trisphosphate (IP_3_) is a second messenger that induces the release of Ca^2+^ from the endoplasmic reticulum (ER). The IP_3_ receptor (IP_3_R) was discovered as a developmentally regulated glycol-phosphoprotein, P400 (Liu et al. 2023). IP_3_ has been found to release Ca^2+^, but it also releases IRBIT (IP_3_R-binding protein released with IP_3_). IRBIT is a pseudo-ligand for IP_3_ that regulates the frequency and amplitude of Ca^2+^ oscillations through IP_3_R (Song et al. 2019). Of note, IP3 inhibits autophagy through the activation of endoplasmic reticulum (ER) IP3 receptors which induce the release of Ca^2+^ leading to the stimulation of the calpain pathway which inhibits autophagy (Chiurillo et al. 2020). However, the reduction of IP3 level reduces ER Ca^2+^ with subsequent activation of autophagy through the AMPK-dependent pathway (Chiurillo et al. 2020). As well, IP3 receptors inhibit autophagy through inhibition of Beclin-1 (Vicencio et al. 2009).

#### SIRT1 signaling pathway

Silent information regulator 1 (SIRT1), a nicotinamide adenine dinucleotide-dependent deacetylase, is a member of the evolutionarily highly conserved superprotein family, which is located in the nucleus and cytoplasm. It can deacetylate protein substrates in various signal transduction pathways to regulate gene expression, cell apoptosis, and senescence, and participate in the process of neuroprotection, energy metabolism, inflammation and the oxidative stress response in living organisms (Batiha et al. 2023). It has been shown that the SIRT1 protein is required for the activation of autophagy (Owczarczyk et al. 2015). In the aging process, SIRT1 expression is reduced leading to a reduction in the activity of autophagy (Miyamoto 2019). The extension of lifespan has been linked to the efficient maintenance of autophagic degradation, a process that declines during aging. Interestingly, recent observations have demonstrated that SIRT1 regulates the formation of autophagic vacuoles, either directly or indirectly through a downstream signaling network (Owczarczyk et al. 2015). The interactions of SIRT1 with the FoxO and p53 signaling can also regulate both the autophagic degradation and lifespan extension emphasizing the key role of autophagy in the regulation of lifespan (Owczarczyk et al. 2015).

**Fig. 3:**
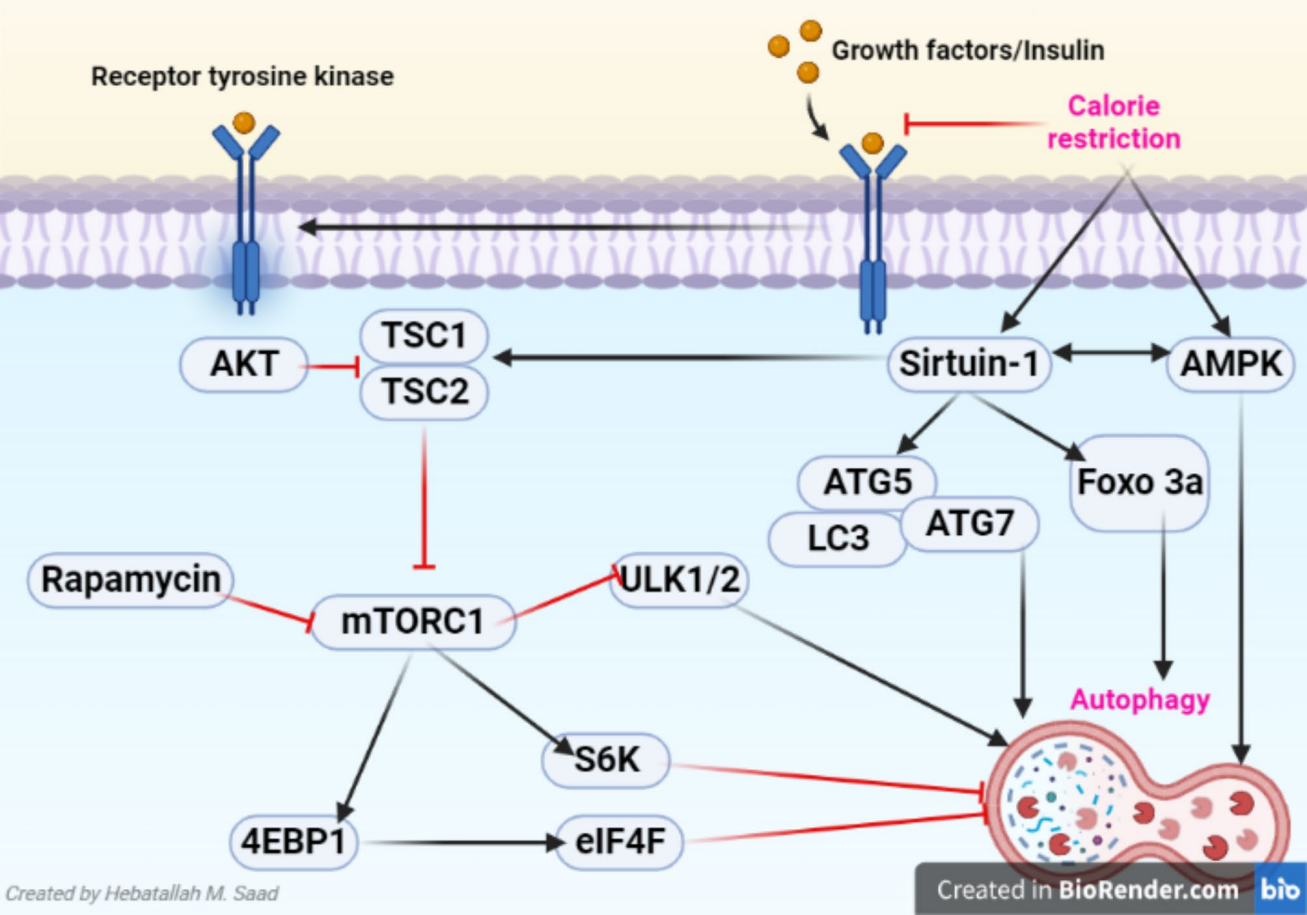
**Regulation of autophagy**: Growth factors like insulin-like growth factor (IGF) through activation of IGF receptors (IGFRs) enhence the activity of tyrosine kinase and Aκt leading to activation of the mTOR pathway and inhibition of autophagy

These findings indicated that different proteins are engaged in the modulation of autophagy which is highly dysregulated in aging (Luo et al. 2020) (Fig. 3).

### Function of autophagy

Autophagy is mainly cytoprotective and protects different organ systems in the body including the CNS, liver, kidney, and heart (Tang et al. 2015). As well, autophagy promotes life span and has anti-aging effects (Luo and Qin 2019). The key role of autophagy is through the induction of derivative pathways for misfolded proteins and neurodegenerative associated proteins such as tau protein and α-Syn (Rana et al. 2021). In addition, autophagy promotes mitochondrial function and prevents the development and progression of mitochondrial dysfunction due to oxidative stress (Rocha et al. 2020). Impaired autophagic processes in neurons lead to inadequate homeostasis and neurodegeneration such as Alzheimer’s disease (AD), Parkinson’s disease (PD), Huntington’s disease, and amyotrophic lateral sclerosis (AlAnazi et al. 2023; Alnaaim et al. 2023). Therefore, autophagy is an essential process for all cells including neurons which mainly depend on autophagy in regulating homeostasis.

## Role of autophagy in epilepsy

It has been suggested that autophagy prevents the development and progression of epilepsy through the regulation of the balance between inhibitory GABA and excitatory glutamate (Qi et al. 2011). In addition, dysfunctional autophagy occurs in epilepsy, mainly caused by an imbalance between excitation and inhibition in the brain (Qi et al. 2011). Ras-related protein Rab-26 (Rab26) and ATG16L regulate neurotransmitter release through autophagy. Rab26 localizes on synaptic vesicles and is preferentially oligomerized to its GDP-bound form, which binds to ATG16L. This complex links synaptic vesicle clusters to the autophagy pathway and may assist in the migration of synaptic vesicles to nearby active sites, involving the regulation of presynaptic autophagy and neuronal activity (Nikoletopoulou and Tavernarakis 2018). It has been shown that an increased ratio of LC3 II to LC3 I and decreased p62 protein levels after epileptic seizure onset, reflect an abnormal increase in autophagic activity (Nikoletopoulou and Tavernarakis 2018). This may trigger chronic hyper-activation of glutamate receptors, slow degradation of GABA-A receptors, and formation of protein aggregates which further exacerbate neuronal damage in epilepsy (Nikoletopoulou and Tavernarakis 2018; Chen et al. 2023). Aberrant autophagy in epilepsy also alters the expression or function of ion channels such as the GABA-A and glutamate receptors, resulting in decreased or increased neuronal excitability. Rapid mobilization of neuronal growth processes that establish new synaptic connections may be disrupted by abnormal autophagy (Nikoletopoulou and Tavernarakis 2018; Chen et al. 2023). However, the underlying molecular mechanisms by which autophagic activity regulates these alterations to promote epileptogenesis remain unclear.

The lack of autophagy in neurons leads to a strong neurodegenerative phenotype and epileptic disorders. The contribution of autophagy in brain physiology and pathology emphasizes the relevancy of the proper control of amino acid levels such as glutamate and GABA in the brain due to their role as neurotransmitters and energy sources (Qi et al. 2011). Autophagy is involved in synaptic homeostasis and regulation of neurotransmitters, thus defective autophagy is linked with a reduction in the activity of certain neurotransmitters mainly GABAergic neurotransmitters (Yin et al. 2018). The diversity and heterogeneity of GABAergic interneurons affect epileptogenesis and hyper-excitability in epilepsy (Marafiga et al. 2021). It has been observed that mTOR/autophagy axis controls synaptic plasticity, vesicular release, and clustering of GABA receptors with the regulation of inhibitory/excitatory balance in the brain (Limanaqi et al. 2020). Elevated activity of mTORC1 is implicated with increased neuronal excitability. The relevance of mTOR regarding temporal lobe epilepsy in animal models and patients has been rising in importance throughout. Excessive mTOR signaling through a mutation in the tuberous sclerosis complex leads to hippocampal hyperexcitability linking mTOR with temporal lobe epilepsy (Bateup et al. 2013). Over-activation of mTOR and associated defective autophagy are linked with the development of seizures in children with focal cortical dysplasia and tuberous sclerosis complex (Yasin et al. 2013). Moreover, the exaggeration of neuronal mTOR also develops in response to the immune system, as autoimmunity induces synaptic alteration and the development of seizures (Limanaqi et al. 2019a, b).

In addition, numerous studies ascribe that autophagy dysfunction is associated with the development of epilepsy. For example, deficiency of Atg7 and Atg18 are associated with spontaneous seizure in animal model studies (Saitsu et al. 2013; Garyali et al. 2014). Particularly, epileptic seizure triggers autophagic dysfunction which further aggravates epileptic seizure in a vicious cycle manner (Gan et al. 2015). Autophagy activity is highly suppressed in the forebrain in patients with tuberous sclerosis complex (McMahon et al. 2012). Deletion of Atg7 in mouse forebrain neurons enhances seizure initiation and progression due to the hypofunction of autophagy (McMahon et al. 2012). Therefore, defective autophagy promotes epileptic seizure in animal and human studies (Yasin et al. 2013; McMahon et al. 2012). The induction of autophagy and autophagy-related proteins like Atg7, LC3, and Beclin-1 by endothelial progenitor cells could be a novel therapeutic strategy in the management of epilepsy (Ali et al. 2019). Furthermore, epilepsy is associated with over-activation of glutamate and excitotoxicity which induce impairment of autophagy in hippocampal neurons (Kulbe et al. 2014). It has been reported that autophagy can attenuate neuronal excitotoxicity (Limanaqi et al. 2019a, b)In this state, the autophagy process seems to be neuroprotective, and autophagy inducers could be effective for both epilepsy and autoimmune disease (Wang et al. 2017).

Despite the protective role of autophagy against epileptogenesis and epilepsy, though its role in SE is perplexing and might be a double-edged sword, may be detrimental or harmful. In animal model epilepsy, autophagy-related protein LC3-II is increased within 6 h following a kainic acid-induced seizure in mice (Shacka et al. 2007). However, the LC3-II level was not correlated with other autophagy-related proteins (Shacka et al. 2007). Another experimental study demonstrated that LC3-II level was increased in parallel with other autophagy-related proteins such as Atg7 in the hippocampus after SE in mice (Otabe et al. 2014). These findings indicated that autophagy activity is correlated with SE. Hence, augmentation of autophagy function in acute seizure and SE could be a compensatory mechanism to restrict neuronal loss and promote rearrangement of neurons and synaptic instability.

In sum, defective autophagy is associated with the induction of epileptogenesis and the development of epilepsy. Increasing autophagy activity in epilepsy and SE may be a compensatory mechanism to reduce neurotransmitter disturbance and synaptic dysfunction. However, the mechanistic role of autophagy in epilepsy is not fully elucidated.

## Mechanistic role of autophagy signaling in epilepsy

### mTOR pathway

Of note, mTOR is a serine-threonine kinase that forms two protein complexes termed mTORC1 and mTORC2 (Wong 2013). As a central check point, mTORC1 senses both internal and external signals such as nutrient and growth factor availability as well as oxidative stress to guide protein synthesis (Wong 2013). As well, mTORC2 is a rapamycin-insensitive complex that contributes to cell survival functions, metabolism, proliferation and actin polymerization. The exact role of mTORC2 in cellular signaling is still unclear. Several neuropathological diseases such as autism, depression and epilepsy have been linked to dysregulation of both complexes (LaSarge and Danzer 2014). It has been shown that the mTOR pathway is involved in the regulation of neurogenesis, synaptic plasticity, neuronal development and excitability (LaSarge and Danzer 2014). Exaggerated mTOR activity is associated with the development of temporal lobe epilepsy, genetic and acquired epilepsy, experimental epilepsy and Lafora disease (Limanaqi et al. 2020). In particular, mTORC1 is mainly involved in epileptogenesis (Wong 2013). Genetic deletion of Tac1 and Pten genes in forebrain neurons in mice induces disinhibition of the mTOR pathway and development of seizure with significant reduction of autophagy (Meng et al. 2013). Thus, inhibition of the mTOR pathway reduces seizure severity through the activation of autophagy (Giorgi et al. 2015). Griffith and Wong (2018) illustrated that inhibition of mTORpathy according to the findings from preclinical and clinical trials may be effective in the management of genetic and acquired epilepsies. Besides, different clinical studies suggest that mTOR pathway inhibitor rapamycin can attenuate and prevent epileptogenesis, and could be effective in treating intractable epilepsy (Ostendorf and Wong 2015). Therefore, autophagy and mTORpathy are considered fundamental pathways in epileptogenesis and epilepsy. Recently, Chen et al. (2023) revealed that pharmacological modulation of autophagy could be a promising therapeutic opportunity in managing epilepsy (Lv and Ma 2020). Likewise, the Rac1 protein which is negatively regulated by the mTOR pathway is implicated in epileptogenesis (Vaghi et al. 2014). Deletion of the Rac1 protein triggers the development of epilepsy by inducing neuronal hyper-excitability and reducing of GABAerigic inhibitory current in the hippocampus through inhibition of autophagy (Vaghi et al. 2014; Pennucci et al. 2016). Okura et al. (2013) found that rapamycin stimulates the expression of Rac1 protein. Consequently, defective Rac1 protein is connected with an exaggerated mTOR pathway and defective autophagy. Seizures generated in the hippocampus have also been related to hyperactive mTOR signaling in a mouse model harboring *PTEN* mutations. Knock-out of PTEN leads to hyperactive mTOR causing seizures generated in the hippocampus, mimicking the epileptic phenotype of focal cortical dysplasia (Matsushita et al. 2016). Therefore, control of excitability by mTOR is crucial to maintain balanced firing of neurons. In genetic mouse models, hyper-activation of mTOR signaling due to loss of the upstream regulators phosphatase and tensin homolog (PTEN) or tuberous sclerosis complex (TSC) has been associated with cortical malformations and the development of epilepsy (Wong and Crino 2012). In addition, mTOR hyper-activation due to *Pten* deletion has been shown to cause epilepsy (Wong and Crino 2012). Future studies are needed to evaluate *Pten*-negative and whether this correlates with the levels of mTOR hyperactivity and epilepsy severity. Thus, mTOR hyperactivity is linked with disturbances of different molecular signaling which involved in epilepsy.

### Glycogen synthase kinase 3

Glycogen synthase kinase 3 (GSK3) is a threonine/serine kinase enzyme that regulates different cellular pathways. GSK3 refers to two paralogs that are commonly referred to as isoforms, GSK3α and GSK3β (Lin et al. 2020). Over-expression of GSK3 plays an integral in the inhibition and attenuation of the mTOR pathway (Ka et al. 2014). However, both mTOR and GSK3 upstream are the negative regulators of autophagy leading to failure of autophagy as in Lafora disease which is an autosomal recessive epilepsy characterized by resistance epilepsy and dementia (Lohi et al. 2005). Both GSK-3α and GSK-3β are in activated states during the most active phase of cell migration. In addition to having a positive control or permissive, rather than negative, function in cell migration, GSK-3 appears to act upstream of the small GTPases ADP-ribosylation factor 6 (ARF6) and Rac1. Inhibition of GSK-3 reduces activation of Rac1 and migration of cells (Park et al. 2023). Defective Rac1 protein is associated with increasing activity of the mTOR pathway and subsequent inhibition of autophagy (Matsushita et al. 2016). Therefore, GSK-3 inhibits autophagy either directly or indirectly via attenuation of Rac1. GSK3 is implicated in the pathogenesis of AD and epilepsy (Lin et al. 2020). In addition, seizure promotes expression of GSK3 which further exacerbates AD neuropathology (Lin et al. 2020). Therefore, GSK-3 represents an attractive target of interference in AD-associated seizures, such that inhibiting the activity of GSK-3 might have therapeutic potential in ameliorating epileptic seizures in AD. The expression level of GSK-3β and collapsin-responsive mediator protein-2 (CRMP-2) were raised after seizure induction. The alteration of the expression level of GSK-3β and CRMP-2 after seizure induction proposes that GSK-3β and CRMP-2 are crucial for epileptogenesis (Lee et al. 2012a, b).

### AMP-activated protein kinase

AMP-activated protein kinase **(**AMPK**)** is a central regulator of energy homeostasis, which coordinates metabolic pathways and thus balances nutrient supply with energy demand (Ahmad et al. 2020). Because of the favorable physiological outcomes of AMPK activation on metabolism, AMPK has been considered to be an important therapeutic target for controlling human diseases including metabolic syndrome and cancer (Ahmad et al. 2020). Preclinical findings revealed that AMPK the brain from seizure-induced neuronal death through induction of Bcl-2–modifying factor (Bmf) normally interacts with the cytoskeleton, but upon certain cellular stresses, such as loss of extracellular matrix adhesion or energy crisis, Bmf relocalizes to mitochondria, where it can promote Bax activation and mitochondrial dysfunction (Moran et al. 2013). In addition, AMPK regulates neuronal activation during SE, and prevents neuronal injury (Moran et al. 2013). Moreover, AMPK which induces autophagy by inhibition of the mTOR pathway is also intricate in epileptogenesis and epilepsy. It has been shown that AMPK agonist metformin can reduce seizure severity in animal model studies (Sanz et al. 2021). Singh et al. (2018). AMPK improves synaptic plasticity and modulates long-term potentiation preventing neuronal hyper-excitability and epilepsy (Potter et al. 2010). Intermittent caloric restriction induces the expression of autophagy and AMPK signaling which attenuate resistance epilepsy (Yuen and Sander 2014). Hence, the induction of autophagy by AMPK activators can reduce epileptogenesis and the progression of epilepsy.

### Autophagy-related proteins

It has been shown that autophagy-related proteins are intricate in epileptogenesis and the development of epilepsy. For example, the inactivation or deletion of Atg7 which is an activator of autophagy triggers recurrent seizures and the development of epilepsy in mice (McMahon et al. 2012). However, previous studies revealed that deletion of both Atg5 and Atg7 resulted in neurodegeneration without evidence of epilepsy (Hara et al. 2006; Komatsu et al. 2006). Autophagy-related protein 5 (ATG5) is one of the key genes for the regulation of the autophagy pathway. A clinical study observed that ATG5 gene polymorphisms Chinese population are linked with epilepsy (Zhang et al. 2021). Subgroup analysis showed a highly significant association of rs510432 with late-onset epilepsy, and rs548234 were associated with the susceptibility to temporal lobe epilepsy (Zhang et al. 2021). Of interest, neurodegenerative diseases mainly AD is commonly associated with epilepsy even in the early stages (Vossel et al. 2013). The interaction between apoptosis-related protein Bcl-2 and Beclin-1 (Atg6) is dysregulated during epileptic seizure (Li et al. 2018). An experimental study showed that Beclin-1 and LC3 levels were increased, whereas Bcl-2 level was reduced within 48 h following seizure in rats (Li et al. 2018). This finding indicates that autophagy is activated following epilepsy. Also, a Beclin-1-interacting protein Ambra1 is dissociated from Bcl-2 following autophagy induction leading to the activation of autophagy (Li et al. 2018). Therefore, activation of autophagy following epilepsy could be a compensatory mechanism to limit neuronal loss. Suppression of autophagy promotes seizure and neuronal apoptosis (Mao et al. 2019). In addition, different preclinical studies illustrated that LC3II/LC3I ratio and Beclin-1 expression were augmented in animal model epilepsy (Dong et al. 2013; Wang et al. 2019a, b, c). Remarkably, induced autophagy in response to oxidative stress contributes to neuronal cell deaths after seizure in animal model study (Cao et al. 2009). Inhibition of oxidative stress by antioxidants considerably attenuates autophagic response in pilocarpine-induced epilepsy (Cao et al. 2009). These observations indicated that autophagy activity in epilepsy is altered in response to the effect of oxidative stress.

### Phospholipase D

Phospholipase D (PLD) isoenzymes including PLD1 and PLD1 have basic cell functions such as vesicular trafficking as well as in brain development. They hydrolyzed phosphatidylcholine to generate phosphatidic acid and free choline (Nackenoff et al. 2021). PLD participates in receptor endocytosis as well as in exocytosis of neurotransmitters where PLD seems to favor vesicle fusion by modifications of the shape and charge of lipid membranes (Tanguy et al. 2020). Many mitogenic factors, including neurotransmitters and growth factors, activate PLD in neurons and astrocytes. Activation of PLD downstream of protein kinase C seems to be a required step for astroglial proliferation (Nackenoff et al. 2021; Tanguy et al. 2020). The post-natal increase in PLD activities concurs with synaptogenesis and myelinogenesis in the brain, and PLD is involved in neurite formation. In the adult and aging brain, PLD activity has antiapoptotic properties suppressing ceramide formation. Increased PLD activities in acute and chronic neurodegeneration as well as in inflammatory processes are evidently due to astrogliosis and may be associated with protective responses of tissue repair and remodeling (Tanguy et al. 2020). It has been shown that PLD inhibits autophagy through activation of mTOR, inhibition of AMPK and inhibition of the interaction between Beclin-1 and vacuolar sorting protein 34 (Vps34) (Yin et al. 2018; Mukhopadhyay et al. 2015; Jang et al. 2014). PLD activity is augmented within neurons and astrocytes in animal epilepsy models (Kim et al. 2004). PLD1 and PLD2 have unique pathophysiological functions in the rat hippocampus after kianic acid-induced seizures (Kim et al. 2004). Of interest, temporal lobe epilepsy-induced seizure provokes BBB injury through the activation of phospholipase A2 (PLA2) (Bera et al. 2022). In vitro study showed that expression of PLA2 was augmented in the hippocampus of animals and humans with temporal lobe epilepsy (Bera et al. 2022). PLA2 triggers autophagy in patients with gouty arthritis (Fu et al. 2023). Similarly, in vitro study revealed that PLA2 promotes the activation of macrophage autophagy (Qi et al. 2011). Thus, activation of PLD and PLA2 during seizures may induce significant alteration of autophagy function. However, PLD is mainly involved in the inhibition of autophagy by activating the mTOR signaling pathway, and the inhibition of AMPK and autophagic proteins.

### SIRT1

SIRT1 is an essential protein required for activation of autophagy (Owczarczyk et al. 2015). Reduction of SIRT1 expression during aging process leads to a decrease in the activity of autophagy (Miyamoto 2019). SIRT1 regulates the formation of autophagic vacuoles via a downstream signaling network (Owczarczyk et al. 2015; Miyamoto 2019). The possible role of SIRT1 in epileptogenesis is complex, as it increased following induced SE in mice. Inhibition of SIRT1 by specific inhibitors did not affect the frequency, duration, and severity of SE in the animal model study without exacerbation of epileptic seizure (Hall et al. 2017). Previous studies indicated that SIRT1 expression was increased in epileptic patients and experimental models of epilepsy within 1 h (Chen et al. 2013; Brennan et al. 2016). SIRT1 expression was reduced after 24 h from SE (Wang et al. 2016). The possible protective role of SIRT1 against the development of epileptogenesis and progression of epilepsy is related to different mechanisms including activation of autophagy and melioration of mitochondrial dysfunction which are induced by epilepsy. In addition, SIRT1 promotes peroxisome proliferator-activated receptor gamma co-activator- 1 alpha (PC-1α) which reduces mitochondrial dysfunction and prevents the progression of ROS (Wang et al. 2015). Similarly, SIRT1 activates forkhead box O3 (FOXO3) which activates autophagy and promotes cell survival (Giannakou and Partridge 2004). SIRT1 also inactivates p53 which is involved in neuronal cell death (Kim et al. 2007). Therefore, increasing expression of SIRT1 following SE seems to be neuroprotective by inducing autophagy and other neuroprotective signaling pathway.

### FOXO3 signaling pathway

FOXO3 is a specific transcription factor involved in the generation of reactive oxygen species (ROS) and neuronal apoptosis (Hagenbuchner et al. 2012). FOXO3 can reduce the accumulation of ROS by activating mitochondrial antioxidant enzymes (Olmos et al. 2013). In vitro study demonstrated that a purified mitochondrial fraction from animal and human brains mainly in hippocampal neurons contains a large concentration of FOXO3 in response to epilepsy (Caballero-Caballero et al. 2013). Augmentation of FOXO3 is associated with epileptic severity and temporal lobe epilepsy (Caballero-Caballero et al. 2013). Expression of neuronal mitochondrial FOXO3 was increased in patients with temporal lobe epilepsy as compared to the controls (Caballero-Caballero et al. 2013). It has been reported that FOXO3 induces the activation of autophagy in neural stem cells (Audesse et al. 2019). In response to starvation, FOXO3 expression is upregulated leading to the induction of autophagy by promoting the formation of autophagosomes, and FOXO3 knockdown reduces autophagosomes in skeletal muscles (Mammucari et al. 2007). A recent study suggests that the ketogenic diet could be highly effective in the management of resistance epilepsy through the modulation of neurotransmitters and induction of FOXO3 and autophagy (Ko et al. 2022). Moreover, FOXO3 has a neuroprotective role and reduces seizure severity in acute CNS toxicity by regulating oxidative stress (Zhang et al. 2020). Besides, the brain is highly susceptible to the effect of oxidative stress due to extreme metabolic demand and oxygen consumption. Excessive production of ROS and development of oxidative stress enhance neuronal hyper-excitability and initiation of epileptogenesis and seizure (Geronzi et al. 2018). However, the role of oxidative stress is not similar in all types of epilepsy and seizure models (Shin et al. 2011). Oxidative stress is regarded as one of the essential mechanisms in the initiation and progression of epilepsy (Aguiar et al. 2012). In addition, epilepsy and SE induce cellular injury through the induction of oxidative injury of DNA and membrane lipids and protein by reducing ATP production (Liang and Patel 2006). Different studies demonstrated that mitochondrial oxidative stress was increased in chronic epilepsy and following SE (Liang and Patel 2006; Liang et al. 2000). Kainic-acid-induced seizure induces hippocampal mitochondrial dysfunction and oxidative stress in the animal model study (Shin et al. 2011). These observations proposed a mutual feedback loop between epilepsy and oxidative stress in relation to the FOXO3 signaling pathway. Therefore, higher expression of FOXO3 and associated activation of autophagy in epilepsy might be a compensatory mechanism to mitigate oxidative stress and linked neuronal injury.

### p53 gene

Of note, the p53 gene is a transcription factor that regulates the mTOR pathway and autophagy, is involved in the induction of apoptosis in response to hypoxia and oxidative stress (Morrison and Kinoshita 2000). Oxidative stress can induce the expression of the p53 gene during epileptic seizures (Tong et al. 2020; Chuang 2010). Importantly, p53 expression is correlated with epileptic seizure in animal model studies (Culmsee et al. 2001) and temporal lobe epilepsy (Engel et al. 2007). Consequently, p53 inhibitors could be effective in the management of resistance epilepsy. However, loss of p53 exacerbates epilepsy following SE (Engel et al. 2010). The experimental study demonstrated that kainic acid-induced seizure was more severe in p53-deficient mice as compared to the wild type (Engel et al. 2010). Therefore, the use of p53-inhibitors for resistance epilepsy should be revised. Autophagy inhibits p53 expression, and deficiency of Atg7 promotes p53-dependent apoptosis (Lee et al. 2012a, b). Thus, autophagy seems to play a critical role in the attenuation of epilepsy by inhibiting the expression of p53.

### IP3/IMPase

Inositol monophosphatase-1 (IMPase-1) is primarily responsible for releasing free *myo*-inositol from several of its inositol monophosphates after brain receptor stimulation, or from glucose through the de novo pathway (Chiurillo et al. 2020). IMPase-1 is a pivotal enzyme in the brain inositol signaling system and appears to be the key enzyme required for the replenishment of brain inositol implicated in neuronal signaling (Chiurillo et al. 2020). The effect of inositol-deficient food supports the role of inositol depletion in the effects of lithium on pilocarpine-induced behavior and epilepsy. However, the relevance of this behavior to other more mood-related effects of lithium is not clear (Shtein et al. 2015). Inhibition of IMPase by phenytoin provides new insights regarding the mechanism of action of phenytoin in the pathophysiology of either bipolar disorder or epilepsy (Mariotti et al. 2010). Indeed, IP3 inhibits autophagy via the stimulation of ER IP3 receptors (Chiurillo et al. 2020). In addition, IMPase inhibits autophagy (Yang et al. 2021) therefore inhibition of IP3/ IMPase could improve neuronal autophagy. Henceforth, lithium is suggested to be effective in treating resistance epilepsy by inhibiting the IP3/ IMPase axis and induction of autophagy (Bojja et al. 2022).

**Fig. 4:**
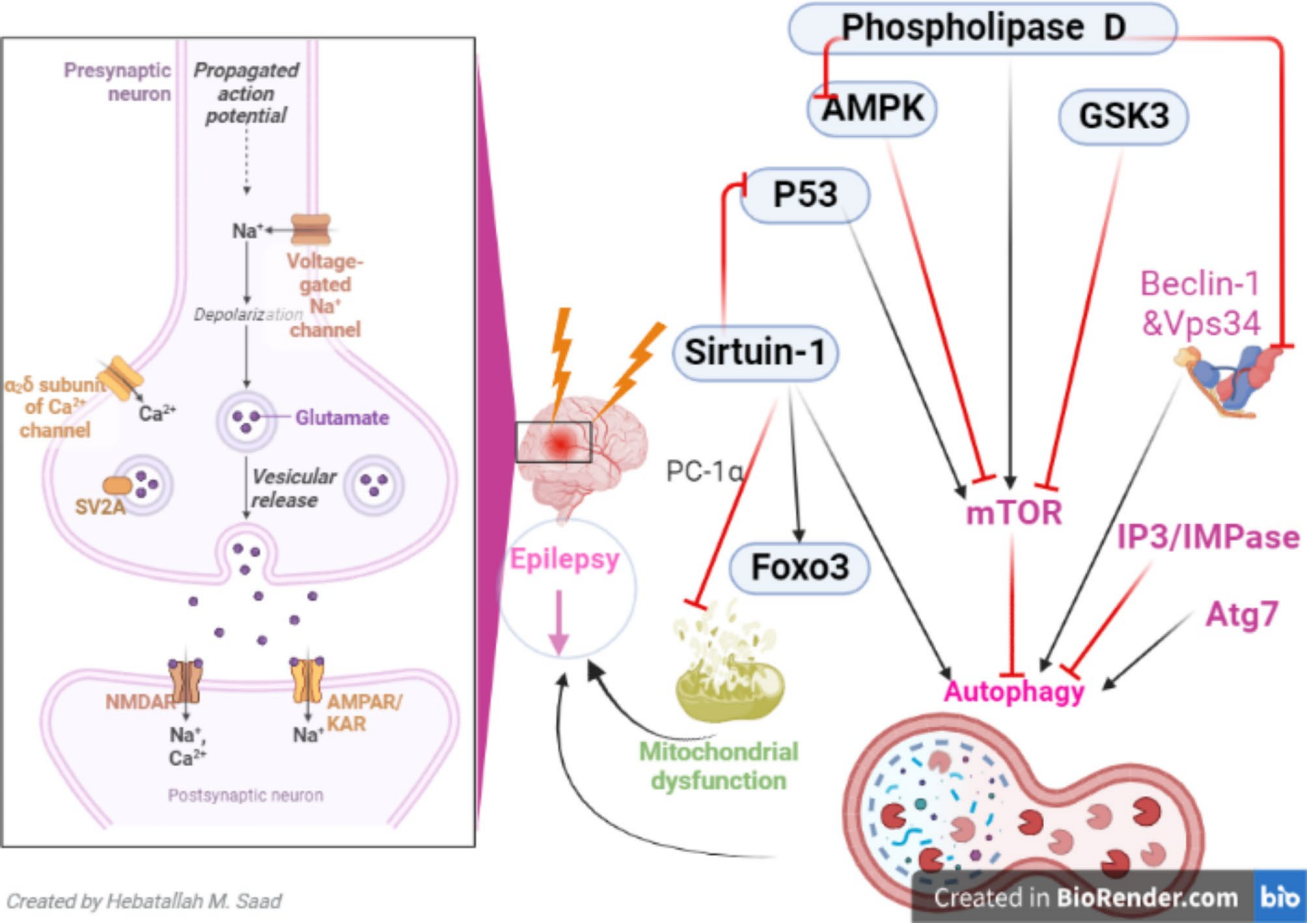
**Autophagy and epilepsy.** Sirtuin-1 (SIRT1) activates FOXO3, and autophagy, inhibits P53 and promotes peroxisome proliferator-activated receptor gamma co-activator 1 alpha (PC-1α) which reduces mitochondrial dysfunction. Phospholipase D inhibits autophagy through activation of mTOR, inhibition of AMPK and inhibition of the interaction between Beclin-1 and vacuolar sorting protein 34 (Vps34). P53 and IP3 inhibit autophagy through the activation of the mTOR pathway and endoplasmic reticulum (ER) IP3 receptors, respectively. Additionally, autophagy is activated by Atg7 and glycogen synthase kinase 3 (GSK3). GSK3 inhibits the mTOR pathway which is implicated in epileptogenesis

Taken together, these judgments highlighted that dysregulation of autophagy signaling is involved in epilepsy (Fig. 4). However, the direct responsibility of autophagy in epilepsy needs to be verified by additional preclinical and clinical studies.

## Autophagy activators in Epilepsy

### Lithium

Lithium is used primarily for long term prophylactic treatment of bipolar disorders to prevent further manic and depressive recurrences. In this indication, lithium remains the first-line treatment. However, lithium has other clinical effects that may be partially independent of each other (Bojja et al. 2022). Lithium is a natural element discovered in 1817 and used as a mood-stabilizing agent in the management of bipolar disorders. Lithium has pro-convulsive, anti-convulsive and neuroprotective properties (Bojja et al. 2022). Of note, lithium induces sustained increases in cerebral gray matter volume in patients with bipolar disorders and these changes are related to the therapeutic efficacy of lithium (Lyoo et al. 2010). In pilocarpine-induced seizure in mice, administration of lithium at a low dose (10 mg/kg) reduces seizure severity, though a high dose of lithium (40 mg/kg) increases seizure severity (Jiang et al. 2018). These findings provide a framework for further investigations of the underlying electrophysiological mechanisms of lithium-induced imbalances in excitatory and inhibitory neural circuits that regulate seizure activity (Jiang et al. 2018). Furthermore, clinical studies reported that long-term use of lithium is associated with myoclonic, tonic-clonic and non-convulsive SE (Shorter 2009; Kanner 2016). Conversely, a large observational study showed no clinical evidence for seizure occurrence (Lyoo et al. 2010). Of note, lithium promotes autophagy by inhibiting a negative regulator IP3 (Sarkar et al. 2005). Lithium induces autophagy, and thereby, enhances the clearance of autophagy substrates such as α-Syn. The autophagy-enhancing properties of lithium were mediated by inhibition of IMPase leading to inositol depletion. This, in turn, decreased IP_3_ levels. This novel pharmacologic strategy for autophagy induction is independent of mTOR and may help treatment of various neurodegenerative diseases (Sarkar et al. 2005). As well, lithium has direct and indirect inhibitory effects on the expression and activity of GSK3β a negative regulator of autophagy (Motoi et al. 2014). However, preclinical studies observed that high-dose lithium inhibits autophagy while low dose activates autophagy (Shimada et al. 2012). Thus, the lithium-specific dose has an epileptic activity by promoting autophagy through mTOR-dependent and independent pathways.

### Rapamycin

Although rapamycin, the oldest inhibitor of mTOR, was discovered more than 30 years ago, changed interest in this pathway is evident in the numerous rapalogs recently developed. These newer agents borrow from the structure of rapamycin and, although there are some pharmacokinetic differences, they appear to differ little in terms of pharmacodynamic effects and overall tolerability (Li et al. 2014). Rapamycin is a macrolide antibiotic and clinically known as sirolimus. Rapamycin was shown to have multiple actions including anti-proliferative and immunosuppressive effects through modulation of mTOR and FK506-binding protein (Li et al. 2014). It has been shown that autophagy inducers such as rapamycin were effective against epileptogenesis and epilepsy in the acute and chronic phases of kainic acid-induced seizure in mice (Zeng et al. 2009). Direct infusion of rapamycin into the hippocampus following SE in mice for 2 months did not prevent the seizure, as it recurred following the withdrawal of rapamycin (Zeng et al. 2009). This finding indicates that rapamycin has an anti-epileptostatic effect as it interferes with epileptogenesis but not epilepsy (Ryther and Wong 2012). However, chronic administration of rapamycin in rats following SE prevents the occurrence of epileptic seizures through the maintenance of BBB integrity (Vliet et al. 2012). However, the recurrence of epileptic seizures following the discontinuation of rapamycin in rats was not mentioned by the authors.

Evidence from clinical studies indicated that rapamycin is effective in patients with tuberous sclerosis complex, and could be as an adjuvant treatment with AEDs (Zhao et al. 2023). Zhao et al. (2023). Rapamycin mainly inhibits mTORC1 because of the higher sensitivity of this complex to the effect of rapamycin. However, mTORC2 is more resistant to the effect of rapamycin which needs long-term duration to be inhibited (Chong et al. 2012). Rapamycin’s anti-epileptic mechanism is related to its anti-inflammatory and immunomodulatory effects which attenuate the inflammatory reactions induced by SE (Broekaart et al. 2017). It has been shown that rapamycin and other mTOR inhibitors attenuate T cell migration and development of neuroinflammation which trigger epilepsy and SE in different autoimmune disorders like new-onset refractory SE and encephalitis-induced seizure (Shimada et al. 2013; Crino 2019). In particular, rapamycin by inactivation of mTOR inhibits the excitatory circuit in the dentate gyrus and retard the formation of mossy fiber following SE. Similarly, rapamycin reduces the frequency of excitatory post-synaptic current and epileptiform activity in mice with experimental temporal lobe epilepsy (Tang et al. 2012). Interestingly, rapamycin was highly effective against tuberous sclerosis complex-induced seizure in animal and human studies (Goldstein and Hauptman 2021; Zou et al. 2014). Rapamycin reduces seizure frequency in patients with tuberous sclerosis complex by 25% and also reduces the use of AEDs (Zou et al. 2014). A recent clinical study on tuberous sclerosis complex patients with resistance epilepsy revealed that prolonged use of rapamycin for six months reduced seizure frequency by 56.25% (Sadowski et al. 2022). Moreover, rapamycin is regarded as a potent activator of the FK506-binding protein (Li et al. 2014) which inhibits the mTOR pathway. Wang et al. (2019a, b, c) observed that rapamycin attenuates pilocarpine-induced epilepsy in mice (Wang et al. 2019a, b, c). A previous study illustrated that the FK506-binding protein regulates GABAergic neurons and expression of glutamate receptors in astrocytes thereby reducing seizure activity (Sierra-Paredes and Sierra‐Marcuño 2008). Besides, the FK506-binding protein induces autophagy and synergies the effect of antidepressant agents (Gassen et al. 2015). Antidepressant agents induce autophagy by increasing the expression of FK506-binding protein (Gassen et al. 2015). Krueger et al. (2010). Prolonged use of everolimus decrease seizure frequency and severity in different types of resistance epilepsy (Franz et al. 2018). Therefore, rapamycin and everolimus by inhibiting the mTOR pathway, activating the expression of FK506-binding protein and induction of autophagy can attenuate seizure activity and the development of epilepsy.

### Ibuprofen

Ibuprofen is an anti-inflammatory drug belonging to the non-steroidal anti-inflammatory drug (NSAID) (Varrassi et al. 2020). ibuprofen has a neuroprotective and anti-seizure effect by inhibiting cyclooxygenase (COX) enzyme, inflammatory signaling pathways and pro-inflammatory cytokines (Liu et al. 2020). Preclinical findings revealed that ibuprofen reduces the frequency and severity of SE in PTZ-induced seizures in rats (Liu et al. 2020). The potential epileptic of ibuprofen is merely related to its anti-inflammatory effect but could be mediated by inducing autophagy. A preclinical study confirmed that ibuprofen attenuates SE by inducing autophagy in astrocytes as evident by increased levels of autophagy markers (Peng et al. 2019). Moreover, ibuprofen sensitizes CD-44-expressing cells to the effect of Hsp90 through induction of autophagy (Moon et al. 2019). Most NSAIDs are effective in reducing epileptic seizure in the experimental model (Radu et al. 2017) though selective COX2 inhibitors such as celecoxib are more effective than selective COX inhibitors in pilocarpine-induced epilepsy (Radu et al. 2017). A cohort study involved epileptic patients revealed that co-administration of ibuprofen reduces valproic plasma level by 7.5–30.6% within 1 week (Moon et al. 2019). Therefore, the use of ibuprofen in epileptic patients on valproic acid should be used with caution. These findings highlighted that ibuprofen could be effective in the management of epilepsy.

### Metformin

Metformin is an insulin-sensitizing drug used in the management of type 2 diabetes (Al-Kuraishy et al. 2023). Metformin has a neuroprotective effect against different neurodegenerative disorders. Metformin improves cognitive dysfunction in animal models of epilepsy (Yimer et al. 2019). Metformin with caloric restriction in animal model studies reduces seizure risk by increasing AMPK and reducing the mTOR pathway (Rubio Osornio MdC et al. 2018). Metformin has been established to reduce epileptogenesis in animal models (Yang et al. 2017). Experimental studies established that metformin was effective in treating temporal lobe epilepsy and SE in rats (Mehrabi et al. 2018). A systematic review revealed the effectiveness of metformin against epilepsy in animal model studies (Yimer et al. 2019).

**Fig. 5:**
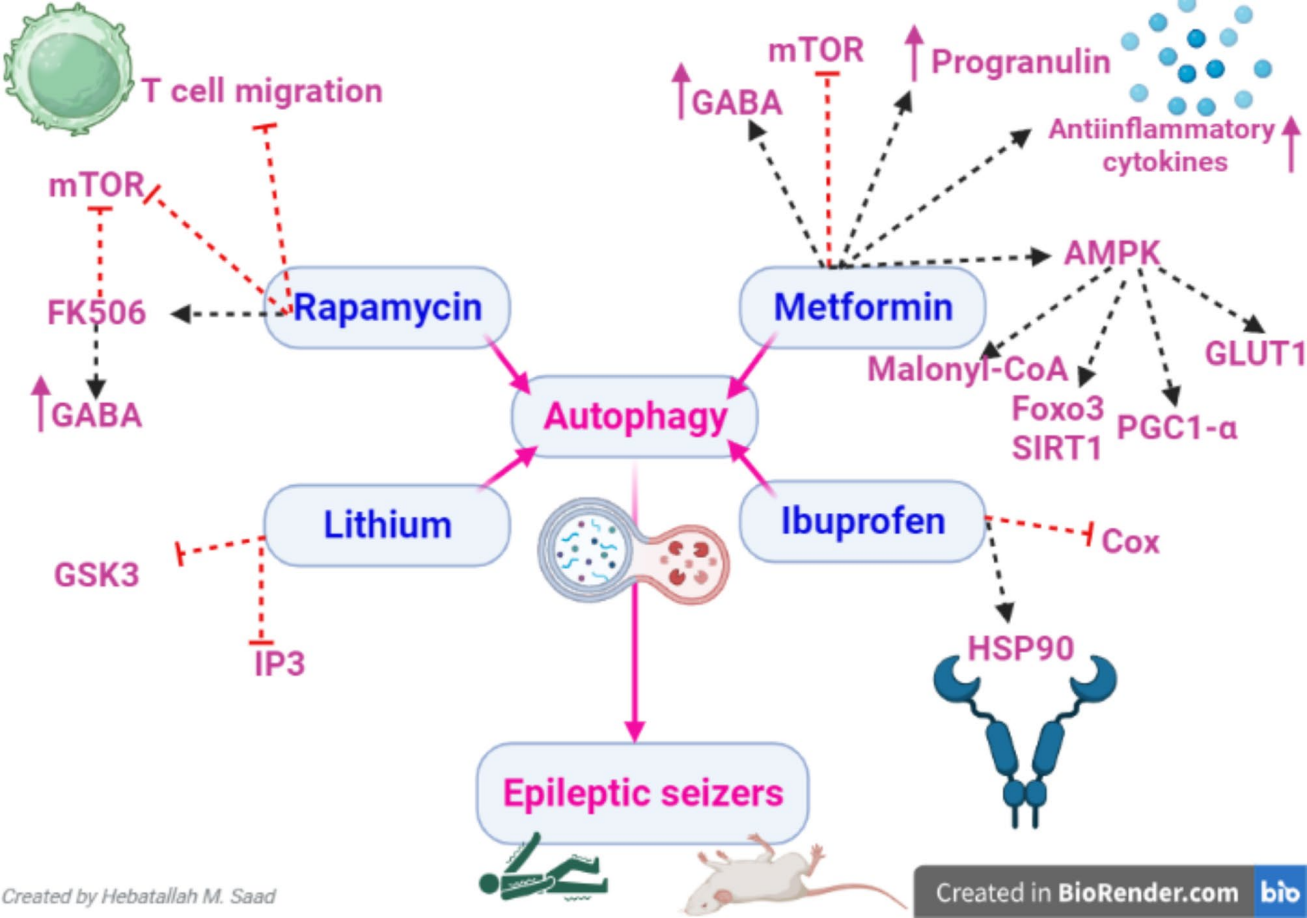
**Mechanistic effects of autophagy inducers in epilepsy**: Rapamycin inhibits mTORC1 and attenuates T cell migration and development of neuroinflammation which trigger epilepsy. Additionally, Rapamycin activates the FK506-binding protein that regulates gamma-aminobutyric acid (GABA) ergic neurons and expression of glutamate receptors in astrocytes thereby reducing seizures. Metformin has anti-seizure activity by activating 5’ AMP-activated protein kinase (AMPK) signaling and inhibiting mTOR pathways which are dysregulated in epilepsy. AMPK improves the expression of glucose transporter 1 (GLUT1) and peroxisome proliferator-activated receptor-γ coactivator 1-α (PGC1α) which improves mitochondrial biogenesis, and upregulates Sirtuin1 (SIRT1), Forkhead box O3 (FOXO3), progranulin and GABA which induce neuroprotection. Ibuprofen has a neuroprotective and anti-seizure effect by inhibiting cyclooxygenase (COX) and heat shock protein 90 (Hsp90) for the induction of autophagy. Lithium inhibits the expression and activity of glycogen synthase kinase-3 beta (GSK3β) and inositol trisphosphate (IP3) which are the negative regulators of autophagy

Furthermore, metformin increases the neuroprotective progranulin and anti-inflammatory cytokines in rats with experimental temporal lobe epilepsy (Vazifehkhah et al. 2020). A randomized clinical trial discovered that metformin reduced seizure frequency in children with tuberous sclerosis complex and Lafora disease (Amin et al. 2021; Burgos et al. 2023). A cohort study involved 18 patients with Lafora disease, 8 treated with metformin, and 10 untreated showed that metformin was effective in reducing seizure severity and frequency (Burgos et al. 2023). These preclinical and clinical findings indicated metformin could be an effective agent against epilepsy mainly the refractory one. AMPK and mTOR are highly expressed in the brain and interconnected mutually in the regulation of energy balance and homeostasis (Li et al. 2019). AMPK which is activated by starvation and metformin activates the catabolic pathway and inhibits the anabolic pathway (Zhang et al. 2016). However, mTOR which is activated by high energy activates the anabolic pathway and inhibits the catabolic pathway (Sangüesa et al. 2019). Activation of AMPK by metformin triggers inhibition of the mTOR pathway either directly or indirectly. In addition, metformin can inhibit mTOR through the AMPK-independent pathway (Lu et al. 2019). Furthermore, AMPK improves the expression of glucose transporter 1 (GLUT1) which is expressed in astrocytes and regulates central glucose homeostasis (Koepsell 2020). Deletion or mutation of astrocyte GLUT1 induces seizures in patients with GLUT1 deficiency syndrome (Koepsell 2020). AMPK enhances glucose uptake and glycolysis of astrocytes by increasing the translocation of membrane GLUT1 (Muraleedharan et al. 2020). However, over-activation of AMPK during brain ischemia has deleterious effects (Ramamurthy and Ronnett 2012). In brain ischemia, AMPK is activated in the astrocytes due to the increase of nitric oxide (NO) which inhibits mitochondrial respiration and promotes glycolysis (Ramamurthy and Ronnett 2012). Moreover, AMPK induces the expression of peroxisome proliferator-activated receptor-γ co-activator 1-α (PGC1α) which improves mitochondrial biogenesis, and upregulates SIRT1 and FOXO3 which induces neuroprotection (Steinberg and Carling 2019; Greer et al. 2007). In addition, AMPK inhibits the synthesis of fatty acids and increases their degradation by increasing the expression of malonyl-CoA which inhibits fatty acid oxidation (Steinberg and Carling 2019). Metformin through an AMPK-dependent pathway inhibits gluconeogenesis in both astrocytes and neurons leading to an increase in glucose flux and increased glycolysis in astrocytes (Shaw et al. 2005; Berthier et al. 2016). Concerning the anti-seizure activity of AMPK, it has been shown that AMPK modulates thalamic spike wave seizure in hypoglycemia-induced absence seizure. In experimental rats, administration of AMPK metformin potentiates seizure network activity via activation of postsynaptic GABA-B in the thalamocortical neurons (Salvati et al. 2022). Metformin like other AEDs such as tiagabine and vigabatrin can trigger absence seizure by increasing the availability of GABA which induces stimulation of GABA-B (Yang et al. 2003). Metformin is effective against temporal lobe epilepsy but exacerbate the absence of seizure (Liu et al. 2006). Particularly, absence seizure is common in children where metformin is rarely used. Therefore, the anti-seizure effect of metformin seems to be identical to the effect of AEDs which are effective for generalized but not for absence seizure.

Furthermore, some types of epilepsy are associated with the up-regulation of the mTOR pathway, and inhibition of this pathway by AMPK activators such as metformin can reduce seizure frequency and severity (Kim et al. 2022). In addition, electroconvulsive shock (ECS) which is used in severe depression can reduce seizure severity through activation of AMPK and inhibition of mTOR which is involved in epileptogenesis (Kim et al. 2022). As well, mTOR inhibitor rapamycin attenuates lipopolysaccharide (LPS)-induced absence seizure in rats through modulation of neuroinflammation (Russo et al. 2014). However, metformin which inhibits the mTOR pathway exacerbates the absence of seizure (Salvati et al. 2022). Therefore, mTOR inhibitors exert different mechanistic pathways against seizure neuro-activity. Particularly, not all mTOR inhibitors are effective in mitigating epileptogenesis (Koene et al. 2019). For example, experimental mTOR inhibitors such as AZD8055 and PF4708671 were shown to be ineffective in mice with epilepsy (Koene et al. 2019). Similarly, vigabatrin which inhibits the mTOR pathway delays but not prevent seizure occurrence in animal model studies (Koene et al. 2019). Furthermore, different preclinical studies confirmed that metformin enhances autophagy (Lu et al. 2021; Bharath et al. 2020; Tomic et al. 2011). Metformin improves autophagy function through modulation of the AMPK/mTOR axis (Lu et al. 2021). As well, metformin regulates mitochondrial function and attenuates aging-induced inflammation by regulating inflammaging and activating autophagy (Bharath et al. 2020). In vitro study demonstrated that metformin inhibits the growth of melanoma by activating autophagy (Tomic et al. 2011). Furthermore, metformin attenuates the development of SE by inducing autophagy (Mohamed et al. 2020). These findings suggest that metformin has anti-seizure activity by activating AMPK signaling and inhibiting mTOR pathways which are dysregulated in epilepsy.

Regarding the safety profile of autophagy activators in epilepsy, both lithium and rapamycin are not appropriate for long-term due to systemic adverse effects such as diabetes inspidus and bone marrow depression caused by lithium and rapamycin respectively (Livingstone and Rampes 2006; Johnson and Kaeberlein 2016). However, long-term adverse effects related to ibuprofen seem to be minor compared to lithium and rapamycin. Remarkably, metformin which also can be used in non-diabetic patients, as it does not cause hypoglycemia, can be used for long-term in patients with generalized epilepsy (Al-Kuraishy et al. 2023; Alrouji et al. 2023). A future perspective regarding the use of autophagy activators in patients with epilepsy should be concerned with the pharmacokinetic and pharmacodynamics interactions between autophagy activators and AEDs. Therefore, clinical trials regarding the combination of autophagy activators with AEDs in different types of epilepsies are warranted.

In sum, autophagy inducers play a critical role in reducing seizure frequency and severity and could be adjuvant treatment in the management of epilepsy (Fig. 5).

## Conclusion

Epilepsy is a neurological disease characterized by repeated seizures. AEDs control epilepsy in about 69%, and one-third of epileptic patients are not controlled by AEDs called refractory epilepsy. Dysregulation of autophagy is intricate in the pathogenesis of epilepsy. Autophagy prevents the development and progression of epilepsy through regulating the balance between inhibitory GABA and excitatory glutamate. Induction of autophagy and autophagy-related proteins like Atg7, LC3, and Beclin-1 might be an innovative therapeutic strategy in managing epilepsy. Despite the protective role of autophagy against epilepsy, though its role in SE is perplexing and might be a double-edged sword, may be detrimental or harmful. Autophagy activators such as rapamycin, metformin and ibuprofen play a critical role in reducing seizure frequency and severity and could be adjuvant treatments in the management of epilepsy. Therefore, autophagy activators could be effective in the management of epilepsy and can be used as adjuvant treatments in the management of resistance epilepsy. Additional preclinical and clinical studies are recommended in this regard.

## Data Availability

Not applicable.

## List of abbreviations

AEDs: Antiepileptic drugs
GABA: Gamma-aminobutyric acid
CMA: Chaperone-mediated autophagy
Vps34: Vacuolar sorting protein 34
IGF: Insulin-like growth factor
AMPK: Adenosine monophosphate protein kinase
PTEN: Phosphate and tensin homology
IMPase: Inositol monophosphatase
SIRT1: Sirtuin-1
mTORC1: mTOR complex 1
GSK3: Glycogen synthase kinase 3
FOXO3: Forkhead box o3
ROS: Reactive oxygen species
NO: Nitric oxide
PGC1α: Peroxisome proliferator-activated receptor-γ coactivator 1-α
LPS: Lipopolysaccharide
NSAID: Non-steroidal anti-inflammatory drug
COX: Cyclooxygenase
